# MicroRNAomes of Cattle Intestinal Tissues Revealed Possible miRNA Regulated Mechanisms Involved in *Escherichia coli* O157 Fecal Shedding

**DOI:** 10.3389/fcimb.2021.634505

**Published:** 2021-02-24

**Authors:** Ou Wang, Mi Zhou, Yanhong Chen, Tim A. McAllister, Graham Plastow, Kim Stanford, Brent Selinger, Le Luo Guan

**Affiliations:** ^1^ Department of Agricultural, Food and Nutritional Science, University of Alberta, Edmonton, AB, Canada; ^2^ Lethbridge Research Centre, Agriculture and Agri-Food Canada, Lethbridge, AB, Canada; ^3^ Research and Innovation Services, University of Lethbridge, Lethbridge, AB, Canada; ^4^ Department of Biological Sciences, University of Lethbridge, Lethbridge, AB, Canada

**Keywords:** cattle, microRNA, gut, *E. coli* O157, super-shedder

## Abstract

Cattle have been suggested as the primary reservoirs of *E. coli* O157 mainly as a result of colonization of the recto-anal junction (RAJ) and subsequent shedding into the environment. Although a recent study reported different gene expression at RAJ between super-shedders (SS) and non-shedders (NS), the regulatory mechanisms of altered gene expression is unknown. This study aimed to investigate whether bovine non-coding RNAs play a role in regulating the differentially expressed (DE) genes between SS and NS, thus further influencing *E. coli* O157 shedding behavior in the animals through studying miRNAomes of the whole gastrointestinal tract including duodenum, proximal jejunum, distal jejunum, cecum, spiral colon, descending colon and rectum. The number of miRNAs detected in each intestinal region ranged from 390 ± 13 (duodenum) to 413 ± 49 (descending colon). Comparison between SS and NS revealed the number of differentially expressed (DE) miRNAs ranged from one (in descending colon) to eight (in distal jejunum), and through the whole gut, seven miRNAs were up-regulated and seven were down-regulated in SS. The distal jejunum and rectum were the regions where the most DE miRNAs were identified (eight and seven, respectively). The miRNAs, bta-miR-378b, bta-miR-2284j, and bta-miR-2284d were down-regulated in both distal jejunum and rectum of SS (log_2_fold-change: −2.7 to −3.8), bta-miR-2887 was down-regulated in the rectum of SS (log_2_fold-change: −3.2), and bta-miR-211 and bta-miR-29d-3p were up-regulated in the rectum of SS (log_2_fold-change: 4.5 and 2.2). Functional analysis of these miRNAs indicated their potential regulatory role in host immune functions, including hematological system development and immune cell trafficking. Our findings suggest that altered expression of miRNA in the gut of SS may lead to differential regulation of immune functions involved in *E. coli* O157 super-shedding in cattle.

## Introduction

*Escherichia coli* (*E. coli*) O157 is a foodborne pathogen that causes severe human disease, such as hemolytic uremic syndrome, bloody diarrhea, and even death (Pennington, 2010). The *E. coli* O157 in the feces of cattle shed into the farm environment can survive in the soil and water for extended periods of time (Maule, 2000) and can cause contamination of vegetables and fruits during planting and irrigation. Furthermore, fecal contamination in the food processing and preparation chain can also lead to the adulteration of meat (Ferens and Hovde, 2011). Cattle shedding >10^4^ CFU *E. coli* O157 per gram of feces are often referred as super-shedders (SS), which have been reported to be responsible for most of the *E. coli* O157 spread into the farm environment (Chase-Topping et al., 2008). However, only a small portion (<10%) of the cattle in a herd have been reported to be SS in a number of studies (Chase-Topping et al., 2008; Munns et al., 2014), suggesting certain individuals have a propensity to become SS. The recto-anal junction (RAJ) of cattle has been suggested to be the primary colonization site of *E. coli* O157 (Naylor et al., 2003), and our recent study revealed that expression of genes involved in immune functions at the RAJs differed between natural SS and non-shedders (NS, cattle negative for *E. coli* O157) (Wang O. et al., 2016). However, the regulatory mechanisms responsible for this differential gene expression in the RAJ of SS *vs* NS remains unclear.

One possibility is that the non-LEE-encoded type III secretive proteins and Shiga toxins produced by *E. coli* O157 may suppress host lymphocyte responses, as suggested by both *in vitro* and *in vivo* studies (Menge et al., 2003; Hoffman et al., 2006; Walle et al., 2013). Another possibility is that the regulatory mechanisms of the transcriptome that lead to immune responses in the gut may differ between SS and NS. One form of transcriptome regulation occurs through non-coding RNAs, especially microRNAs (miRNAs), which have been reported to regulate gene expression in many biological processes (Wahid et al., 2010). In addition, detection of miRNAs in extracellular spaces, such as milk, serum, urine and feces (Weber et al., 2010; Liu et al., 2016), suggests that the functions of miRNAs are not restricted to within cells, but also with extracellular functions associated with functions including immune responses, cell communication and even shaping the gut microbiota (Turchinovich et al., 2012; Liu et al., 2016).

Gene expression is regulated by miRNA through recognition of complementary sequences on transcripts, commonly through binding to the 3′-UTR region (Bartel, 2009; Fang and Rajewsky et al., 2011; Allantaz et al., 2012). Many studies have reported that miRNA expression was associated with host–microbial interaction. Bao et al. (2015) studied whole blood miRNAomes of *Salmonella-* challenged pigs, and suggested that miR-214 and miR-331-3p may regulate host immune responses associated with persistent shedding of *Salmonella*. Others have reported that miRNA-155 influences the presence of *Helicobacter pylori* in mouse gastrointestinal tract by mediating T-cell responses (Oertli et al., 2011). *Mycobacterium tuberculosis* has been reported to interfere with the expression of miR-125b and miR-155 blocking the expression of Tumor Necrosis Factor (TNF) responses in macrophages (Rajaram et al., 2011). Moreover, a recent effort revealed that the expression of miRNAs involved in B-cell functions in the small intestine were associated with beneficial bacterial (*Lactobacilli* and *Bifidobacterium*) populations in the gut of young calves (Liang et al., 2014). Therefore, we hypothesize that the expression of gut miRNAs differs between SS and NS and that it is one of the key regulatory mechanisms of the reported differential gene expression through the gut in response to the *E. coli* O157 colonization and shedding in cattle (Wang O. et al., 2016). We performed miRNA expression profiling on the whole gut of cattle, including the duodenum, proximal jejunum, distal jejunum, cecum, spiral colon, and descending colon using miRNA sequencing to examine whether miRNAs may play a regulatory role in host immune functions that may be associated with the presence of *E. coli* O157 within the digestive tract.

## Materials and Methods

The steers used in this experiment followed the Canadian Council of Animal Care Guidelines, and the protocol was reviewed and approved by the Animal Care Committee of Lethbridge Research Centre, Agriculture Agri-food Canada (Animal Care Committee protocol number: 1120; approved June 2011).

### Super-Shedder Cattle Identification and Intestinal Tissue Collection

The s for identification of SS and tissue collection were as detailed previously (Zaheer et al., 2017). Briefly, fecal samples (50 g each) were collected from RAJ of 400 British × Continental feedlot steers for *E. coli* O157 identification and quantification using plate culturing methods on sorbitol MacConkey agar with 2.5 mg/L potassium tellurite and 0.05 mg/L cefixime. In total, 11 SS were identified with fecal *E. coli* O157 ≥104 CFU per gram of feces. The *E. coli* O157 isolates from CT-SMAC were confirmed using an *E. coli* O157 Latex Test kit (Oxoid Ltd, Basingstoke, Hampshire, UK) and the serotype (*E. coli* O157:H7) was further confirmed with multiplex PCR targeting verotoxin (vt), intimine (eaeA), and H7 flagellin (fliC) following protocols described previously (Gannon et al., 1997). Based on fecal *E. coli* O157 numbers (Wang O. et al., 2016), five SS (out of 11 identified) and five control pen-mates (NS, negative for fecal *E. coli* O157) were used for sampling of intestinal tissue (**Supplementary Table S1**). The fecal *E. coli* O157 of SS were monitored for 4 to 10 days prior to slaughter. All SS were positive for fecal *E. coli* O157 before slaughter (Munns et al., 2014), among them one animal remained SS at slaughter, while the other four were tested positive but not at SS level. NS animals on the other hand, were tested negative for fecal *E. coli* O157 at slaughter. From the slaughtered NS and SS, two 2 cm^2^ of tissues were collected from duodenum, proximal jejunum, distal jejunum, cecum, spiral colon, descending colon, and RAJ (one RAJ sample from a NS was lost during storage) and immediately snap-frozen in liquid nitrogen and stored at −80°C.

### RNA Extraction and miRNA Sequencing

The tissue samples were ground into fine powders in liquid nitrogen prior to RNA extraction. Approximately 100 mg of tissue was used for RNA extraction using a mirVana total RNA Isolation Kit (Ambion, Carlsbad, CA, USA) per manufacturer’s instructions. The RNA integrity was measured using an Agilent 2200 TapeStation (Agilent Technologies, Santa Clara, CA, USA), and the RNA concentration was measured using a Qubit 2.0 Fluorometer (Invitrogen, Carlsbad, CA, USA). The RNA samples with an integrity number (RIN) ≥7.0 and ratio of 28S/18S ≥1.7 were used for miRNA-Seq library construction. For each sample, 1 µg of total RNA was used for library preparation using a TruSeq Small RNA sample preparation kit (Illumina, San Diego, CA) following the manufacturer’s instruction. RNA sequencing was performed using an Illumina HiSeq 2000 (illumina, San Diego, CA, USA) sequencing platform (single end, 1 × 50 bp) at the Genome Quebec Innovation Centre (Montreal, PQ, Canada).

### miRNA Sequencing Data Processing, Functional Analysis, Differential Analysis

All the data were presented as mean ± standard deviation. The miRNA sequencing reads were trimmed, quality filtered, and processed using a web based analysis tool, “sRNAtoolbox” (using default parameters) (Rueda et al., 2015). The read number of detected miRNAs was normalized as counts per million (cpm), and the miRNAs with cpm >1 were defined as expressed miRNAs. The miRNA family conservation was defined according to miRNA family information from TargetScan (release 7.0, 2016) (Agarwal et al., 2015). The miRNA target prediction was performed using PITA (Probability of Interaction by Target Accessibility) (Kertesz et al., 2007) and miRanda (Betel et al., 2008). The targets predicted by both miRanda (matching score > 145, free energy < −10) and PITA (free energy < −10) were used for functional analysis for selected miRNA. The Ingenuity Pathway Analysis^®^ (IPA, IPA^®^, QIAGEN Redwood City, www.qiagen.com/ingenuity) was used for function enrichment, and the Fisher’s exact text implemented by internal functions of IPA was used for the association analysis of miRNA targets and functions, with significance level 5% (Fisher’s exact test p-value 0.05) for selection of enriched functions. The Bioconductor edgeR (Robinson et al., 2010), which fits the read counts to a binomial distribution and estimates the dispersion and determines differential expression by exact test, was used to identify differentially expressed (DE) miRNA. Only miRNAs expressed in >2 of either NS or SS group were used for differential expression analysis, and FDR (false discovery rate) of 10% was used as a cut-off for DE miRNA identification.

### Identification of Potential miRNA Regulation on Target mRNA Expression

To assess the potential for interactions between identified DE miRNAs and predicted target genes, a Spearman’s rank-order correlation analysis between expression of DE miRNAs and all genes that encode predicted target transcripts (gene expression levels are unpublished data, manuscript under review). The correlation analysis was performed using R (version 3.3.2), and only miRNA/transcripts with cpm >1 in at least five animals were used for correlation analysis. The correlation coefficients, *ρ* (rho)-value < −0.6 and p-value <0.05 were considered as significant negative correlation.

### Availability of microRNAome Data

The miRNA-Seq data are available at NCBI Gene Expression Omnibus (GEO) database under accession number GSE96973.

## Results

### miRNAome Profiling of Intestinal Tissues Collected From Beef Steers

**Figure 1:**
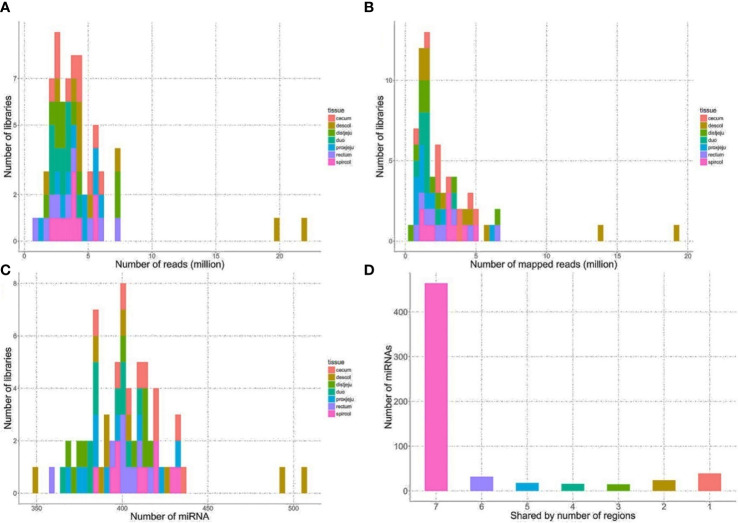
General sequencing results and miRNA mapping results. **(A)** Distribution of numbers of reads generated from HiSeq 2000 sequencing platform. **(B)** Distribution of numbers of reads mapped to known bovine miRNA after quality filtering. **(C)** Distribution of numbers of detected miRNA from the intestinal regions. **(D)** Number of miRNA shared by the intestinal regions, the number on x-axis represents the number of intestinal regions a miRNA is shared by, seven, all intestinal regions; six, six out of seven regions, five, five out of 7 regions, four, four out of seven regions; three, three out of seven regions; two, 2 out of seven regions; one, only detected in one of 7 regions.

**Figure 2:**
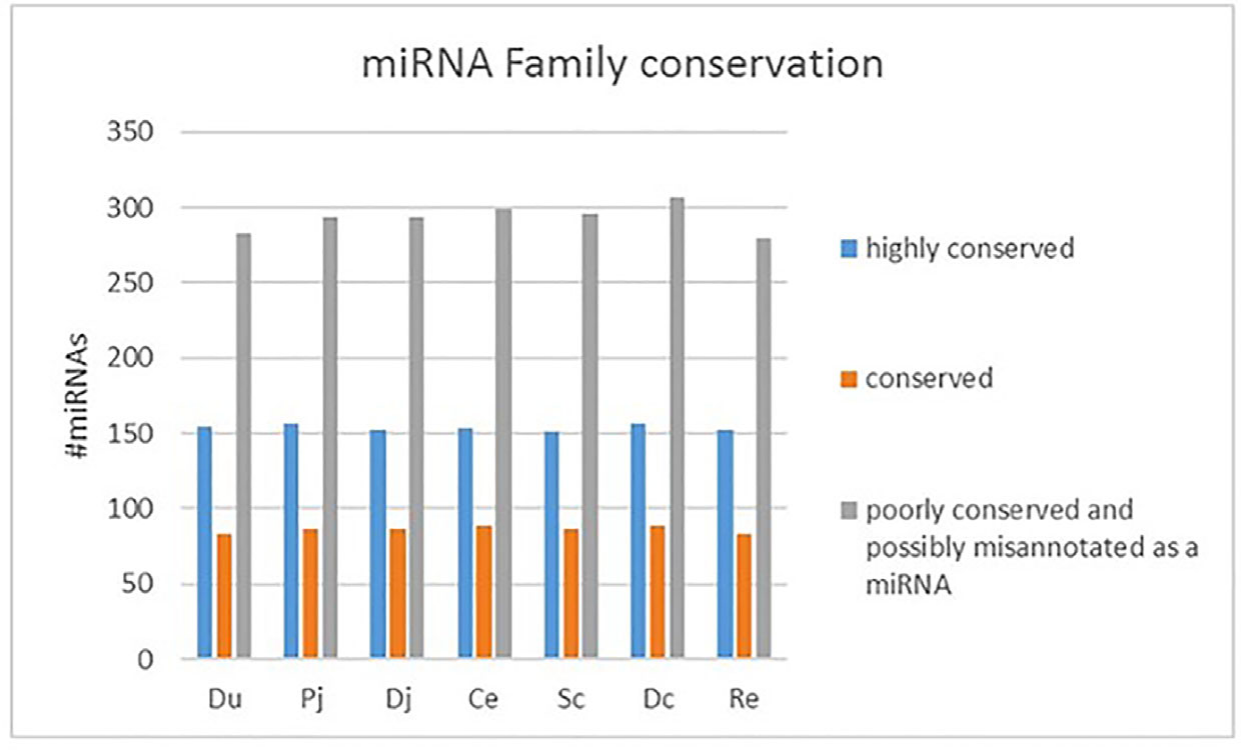
Family conservation of detected bovine miRNAs in each region. Du, duodenum; pj, proximal jejunum; Dj, distal jejunum; Ce, cecum; Sc, spiral colon; Dc, descending colon; Re, rectum.

The sequencing reads were generated from 69 miRNA libraries prepared from intestinal tissues collected at seven different locations, including duodenum, proximal jejunum, distal jejunum, cecum, spiral colon, descending colon, and RAJ. The preliminary miRNA data of RAJ were reported in our previous publication (Wang O. et al., 2016), and we have included in-depth analysis in the current study. The number of sequencing reads ranged from 30 to 75 million for each region (**Table 1**), and the read number for each tissue ranged from 0.9 to 22 million (**Figure 1A**). The number of reads mapped to miRNA ranged from 0.5 to 19 million per sample (**Figure 1B**), and the number of miRNAs detected in each region ranged from 390 ± 13 (duodenum) to 413 ± 49 (descending colon) (**Table 1** and **Figure 1C**). In total, 464 miRNAs were shared by all the intestinal regions (seven regions), while 39 miRNAs were only detected in 1 region (**Figure 1D**). Among the identified miRNAs in each region, the number of highly conserved miRNAs ranged from 151 (spiral colon) to 157 (descending colon), and the number of conserved miRNAs ranged from 83 (duodenum and RAJ) to 88 (cecum and descending colon), while 280 (RAJ) to 307 (descending colon) of detected miRNAs were poorly conserved (defined by TargetScan 7.0 which also claims that certain poorly conserved miRNAs may be sequences misannotated as miRNAs) (**Figure 2**).

**Table 1:** Number of reads generated by Illumina HiSeq 2000 system for miRNA sequencing and average number of miRNAs detected in each intestinal region of beef steers.

Tissue	Number of reads, Million (M)	# identified miRNA
**Duodenum**	30.0	390 ± 13
**Proximal jejunum**	41.1	393 ± 20
**Distal jejunum**	37.7	395 ± 19
**Cecum**	39.9	411 ± 16
**Spiral colon**	38.2	409 ± 15
**Descending colon**	75.4	413 ± 49
**Rectum**** ^a^	33.5	398 ± 15

a Data of rectal tissue were also reported in previous publication (Wang O. et al., 2016).

The bta-miR-143 (48.8%), bta-miR-192 (10.8%) and bta-miR-148a (3.29%) were the three most abundant miRNAs expressed in duodenum, and bta-miR-143 (44.3%), bta-miR-192 (14.1%), and bta-miR-10a (2.7%) were the three most abundant miRNAs expressed in the proximal jejunum. In the distal jejunum, cecum, spiral colon, descending colon, and RAJ, the bta-miR-143 (16.2% in cecum to 59.3% in descending colon), bta-miR-192 (5.4% in spiral colon to 12.5% in cecum), and bta-miR-10b (4.7% in descending colon to 14.6% in cecum) are the three most abundant miRNAs (**Supplementary Table S2**). The relative abundance of bta-miR-143 and bta-miR-192 miRNA accounted for more than 50% of the miRNAs detected in each intestinal region, with the abundance of top 15 miRNAs of each region accounting for >83% of total expressed miRNAs (**Supplementary Table S2**). Among the top 15 most abundant expressed miRNAs, 10 were shared by all seven regions, including bta-miR-143, bta-miR-192, bta-miR-10b, bta-miR-27b, bta-miR-26a, bta-miR-26c, bta-miR-21-5p, bta-miR-148a, bta-miR-22-3p, and bta-let-7f. Functional analysis of these miRNAs indicated that vascular system functions (cardiovascular system development and function, p-values ranged from 4.2E-07 to 3.3E-05) and organismal survival (p-value ranged from 8.9E-07 to 1.6E-04) were frequently enriched (**Table 2**). Also, bta-miR-10b was potentially associated with digestive system development and function (p-value: 1.4E-04), with the bta-miR-148a, bta-miR-192 and bta-miR-22-3p being associated with nervous system development and function (p-value ranged from 2.9E-06 to 3.4E-5) (**Table 2**). In addition, Euclidean distance based hierarchical clustering and PCA analysis indicated that the miRNA expression profiles were not distinct for each of the intestinal regions (**Supplementary Figure S1**).

**Table 2:** Functions enriched for predicted target genes of top 10 most abundant miRNAs that are shared by all the intestinal regions.

miRNA	Function categories*^a^*	p-value*^b^*
**bta-let-7f**	organismal development	7.89E-6
	tissue development	7.89E-6
**bta-miR-10b**	cardiovascular system development and function	1.45E-4
	digestive system development and function	1.45E-4
**bta-miR-143**	organ development	1.28E-5
	organismal survival	1.01E-5
**bta-miR-148a**	cardiovascular system development and function	3.31E-5
	nervous system development and function	4.44E-6
**bta-miR-192**	nervous system development and function	3.45E-5
	organismal development	1.61E-4
**bta-miR-21-5p**	cardiovascular system development and function	4.24E-7
	organismal survival	2.59E-9
**bta-miR-22-3p**	nervous system development and function	2.91E-6
	organismal development	2.22E-6
**bta-miR-26a**	cardiovascular system development and function	8.93E-7
	organismal development	8.93E-7
**bta-miR-26c**	cardiovascular system development and function	1.58E-4
	organismal development	1.58E-4
**bta-miR-27b**	organismal survival	1.96E-9
	tissue development	2.54E-8

(Only the two functions with smallest p-values were shown).

a Functions enriched for the predicted mRNA genes using Ingenuity Pathway Analysis.

b The Fisher’s exact test p-value calculated by Ingenuity Pathway Analysis, and the top two functions with the smallest p-values were shown.

### Differentially Expressed miRNAs Between Non-Shedders and Super-Shedders in Each Intestinal Region

Comparing SS and NS for each region, the number of differentially expressed (DE) miRNAs ranged from one (in descending colon) to eight (in distal jejunum) in the gut tissues, and the log_2_fold-change ranged from −4.5 to 4.5 (**Table 3**). For duodenum (three DE miRNAs), cecum (three DE miRNAs), spiral colon (four DE miRNAs), and descending colon (one DE miRNAs), all identified DE miRNAs were down-regulated in SS. For proximal jejunum (two DE miRNAs) and distal jejunum (eight DE miRNAs), equal numbers of DE miRNAs were up-regulated and down-regulated in SS. For DE miRNAs identified in RAJ, four were down-regulated and three were up-regulated. Four DE miRNAs were shared by more than one intestinal region (**Table 3**): the bta-miR-378b was down-regulated in all regions (log2fold-change ranged from -3.3 to -4.5); bta-miR-2284j was down-regulated (log2fold-change ranged from −2.9 to −4.1) in duodenum, distal jejunum, cecum, spiral colon, and RAJ; bta-miR-2284d (log2fold-change ranged from −2.7 to −2.9) was down-regulated in the duodenum, distal jejunum, spiral colon and RAJ; and bta-miR-99a-5p was up-regulated in the proximal and distal jejunum (log2fold-change were 3.3 and 2.7, respectively) (**Table 3**).

**Table 3:** Differentially expressed miRNAs detected in each region of intestinal tract of beef steers.

Tissue	Differentially expressed miRNA	Log-2-(fold-change)	FDR
	bta-miR-378b	−4.37	<0.01
**Duodenum**	bta-miR-2284j	−3.28	0.02
** **	bta-miR-2284d	−2.90	0.02
**Proximal jejunum**	bta-miR-378b	−4.50	<0.01
** **	bta-miR-99a-5p	3.30	0.04
** **	bta-miR-378b	−3.74	<0.01
	bta-miR-2284j	−4.11	<0.01
	bta-miR-100	3.77	0.01
**Distal jejunum**	bta-miR-2284d	−2.92	0.04
	bta-miR-99b	2.53	0.09
	bta-miR-409a	2.51	0.09
	bta-miR-99a-5p	2.69	0.09
** **	bta-miR-18a	−1.58	0.09
	bta-miR-378b	−3.92	<0.01
**Cecum**	bta-miR-2284j	−3.36	0.02
** **	bta-miR-2904	−2.72	0.02
** **	bta-miR-378b	−3.95	<0.01
**Spiral colon**	bta-miR-451	−1.77	0.01
	bta-miR-2284j	−2.92	0.01
** **	bta-miR-2284d	−2.95	0.01
**Descending colon**	bta-miR-378b	−3.30	0.09
	bta-miR-2284j	−3.76	0.01
	bta-miR-1271	2.59	0.02
	bta-miR-378b	−3.42	0.02
**Rectum**	bta-miR-2887	−3.20	0.05
	bta-miR-211	4.51	0.05
	bta-miR-2284d	−2.72	0.07
	bta-miR-29d-3p	2.23	0.09

(10 tissue samples for each region, including 5 collected from non-shedders and 5 from super-shedders, but only 4 rectal tissues from non-shedders).

For the family conservation of detected DE miRNAs, five miRNAs were defined as poorly conserved (or misannotated as miRNAs), one was conserved, and seven were highly conserved (**Table 4**). Ten of the DE miRNAs were in intergenic regions of the genome, and four of the DE miRNAs were in the intragenic regions (introns) (**Table 4**). Two intronic miRNAs including bta-miR-2284j and bta-miR-2284d, which were likely bovine specific miRNAs, were present in the introns of Glutamate Ionotropic Receptor Kainate Type Subunit 4 (GRIK4) and SWAP Switching B-Cell Complex 70 kDa Subunit (SWAP70), respectively (**Table 4**). The other two intronic miRNAs were highly conserved including bta-miR-1271 and bta-miR-211, which were associated with ADP Ribosylation Factor Like GTPase 10 (ARL10) and Transient Receptor Potential Cation Channel Subfamily M Member 1 (TRRM1), respectively (**Table 4**).

**Table 4:** Differentially expressed miRNA, family conservation, and genomic locations.

miRNAs	Conservation*^a^*	Chromosome	Location
**bta-miR-29d-3p**	2	Chr16	intergenic
**bta-miR-99a-5p**	2	Chr1	intergenic
**bta-miR-100**	2	Chr15	intergenic
**bta-miR-99b**	2	Chr18	intergenic
**bta-miR-18a**	2	Chr12	intergenic
**bta-miR-451**	2	Chr19	intergenic
**bta-miR-211**	2	Chr21	TRPM1*^b^*, intron 7
**bta-miR-1271**	2	Chr7	ARL10*^c^*, intron 2
**bta-miR-409a**	1	Chr21	intergenic
**bta-miR-378b**	−1	Chr15	intergenic
**bta-miR-2904**	−1	Chr2, Chr3	intergenic
**bta-miR-2887**	−1	Chr2, Chr3	intergenic
**bta-miR-2284j**	−1	Chr15	GRIK4*^d^*, intron 3
**bta-miR-2284d**	−1	Chr15	SWAP70*^e^*, intron 2

a Defined by TargetScan (release 7, 2016). 2 = highly conserved across human, mouse, rat, dog, and chicken; 1 = conserved across human, mouse, rat and dog; −1 = poorly conserved across species, potentially bovine specific miRNAs.

b Transient receptor potential cation channel subfamily M member 1.

c ADP ribosylation factor like GTPase 10.

d Glutamate ionotropic receptor kainate type subunit 4.

e SWAP switching B-cell complex 70 kDa subunit.

### Target Gene Prediction and Functional Enrichment for Differentially Expressed miRNAs

The number of predicted targets of DE miRNAs ranged from 37 (bta-miR-99b) to 803 (bta-miR-211) (**Tables 5** and **6**). For the up-regulated DE miRNAs in SS, functions of tissue development (p-value ranged from 5.73E-06 to 1.83E-5), organism development (p-value ranged from 4.42E-07 to 4.64E-06), and nervous system development and functions (p-value ranged from 5.73E-06 to 9.01E-04) were the most frequently enriched (**Table 5**). For down-regulated miRNAs in SS, functions of organism development (p-value ranged from 4.87E-07 to 1.12E-04) and cardiovascular system development (p-value ranged from 1.73E-06 to 2.0E-05) were the most frequently enriched (**Table 6**). In addition, the down-regulated bta-miR-451(log2fold-change: −1.77 in spiral colon) and bta-miR-2284j (log2fold-change ranged from −2.9 to −4.1), as well as up-regulated bta-miR-211 (log2fold-change: 4.5 in RAJ) in SS, were associated with immune functions, such as development of the hematological system (p-value ranged from 1.8E-05 to 1.8E-05) (**Tables 5** and **6**).

**Table 5:** Up-regulated miRNAs in super-shedders and the functions enriched for their predicted target genes.

miRNA	#Target*^a^*	Regions*^b^*	Enriched functions*^c^*	P-value*^d^*
**bta-miR-99a-5p**	40	Pj, Dj	organ development	3.92E-04
			organismal development	3.32E-06
			tissue development	3.32E-06
**bta-miR-100**	47	Dj	nervous system development and function	9.01E-04
			organismal development	4.64E-06
			tissue development	4.64E-06
**bta-miR-409a**	175	Dj	cardiovascular system development and function	4.35E-06
			organismal development	4.35E-06
			tissue development	1.83E-05
**bta-miR-99b**	37	Dj	organismal development	2.67E-06
			tissue development	2.67E-06
			tissue morphology	2.63E-05
**bta-miR-1271**	443	Re	nervous system development and function	5.73E-06
			skeletal and muscular system development and function	5.07E-06
			tissue development	5.73E-06
**bta-miR-211**	803	Re	hematological system development and function	1.78E-05
			nervous system development and function	3.73E-06
			tissue development	3.73E-06
**bta-miR-29d-3p**	521	Re	organ morphology	2.17E-05
			organismal development	4.42E-07
			organismal survival	8.16E-07

a Genes encodes target mRNAs predicted by PITA and miRanda.

b Intestinal regions where the differentially expressed miRNAs were identified. Pj, proximal jejunum; Dj, middle jejunum; Re, rectum.

c Functions enriched for the predicted mRNA genes using Ingenuity Pathway Analysis.

d The Fisher’s exact test p-value calculated by Ingenuity Pathway Analysis, and the top three functions with the smallest p-values were shown.

**Table 6:** Down-regulated miRNAs in super-shedders, and the functions enriched for their predicted target genes.

miRNA	#Target*^a^*	Tissues*^b^*	Enriched functions*^c^*	P-value*^d^*
**bta-miR-378b**	514	Du, Pj, Dj, Ce, Sc, Dc, Re	cardiovascular system development and function	1.73E-06
			connective tissue development and function	5.47E-06
			tissue morphology	1.73E-06
**bta-miR-2284j**	486	Du, Dj, Ce, Sc, Re	cardiovascular system development and function	2.00E-05
			organismal development	1.12E-04
			tissue development	3.07E-05
**bta-miR-2284d**	696	Du, Dj, Sc, Re	cardiovascular system development and function	4.87E-07
			organ morphology	4.87E-07
			organismal development	4.87E-07
**bta-miR-18a**	559	Dj	cardiovascular system development and function	1.33E-06
			organ morphology	4.83E-06
			organismal survival	1.54E-07
**bta-miR-2904**	678	Ce	digestive system development and function	4.12E-05
			hematological system development and function	4.31E-05
			organismal development	3.48E-06
**bta-miR-451**	120	Sc	nervous system development and function	3.75E-06
			skeletal and muscular system development and function	2.64E-05
			tissue development	3.75E-06
**bta-miR-2887**	226	Re	hematological system development and function	1.76E-04
			nervous system development and function	6.50E-05
			organismal development	2.28E-05

a Genes encodes target mRNAs predicted by PITA and miRanda.

b Intestinal regions where the differentially expressed miRNAs were identified. Du, duodenum; Pj, proximal jejunum; Dj, middle jejunum; Ce, cecum; Sc, spiral colon; Dc, Descending colon; Re, rectum.

c Functions enriched for the predicted mRNA genes using Ingenuity Pathway Analysis.

d The Fisher’s exact test p-value calculated by Ingenuity Pathway Analysis, and the top three functions with the smallest p-values were shown.

### miRNA and mRNA Correlation and Functional Analyses

For each intestinal region, the correlation analysis between expression of DE miRNAs and expression of all the computationally predicted target genes indicated that only a small number of predicted target genes were negatively correlated with the miRNAs: one (bta-miR-99a-5p, bta-miR-100 and bta-miR-99b in distal jejunum) to 74 (bta-miR-29d-3p in RAJ) of predicted target genes showed correlation with miRNAs (Spearman’s *ρ* < −0.6, and p-value < 0.05) (**Table 7**). The functional enrichment of negatively correlated target transcripts of each miRNA indicated association between the DE miRNAs and immune functions. As shown in **Table 7**, with bta-miR-378b, the number of negatively correlated transcripts ranged from two (in cecum) to 38 (in spiral colon), and in duodenum, proximal jejunum and distal jejunum, the top enriched functions (smallest p-value) were associated with immune functions, including cell-mediated immune responses and hematological system development (p-values < 0.05). In RAJ, two transcripts were negatively correlated with bta-miR-378b, PRDM1 (PR/SET Domain 1) (*ρ* = −0.68, p-value < 0.05) and CYLD (CYLD Lysine 63 Deubiquitinase) (*ρ* = −0.81, p-value < 0.05), which were also associated with humoral and cell-mediated immune responses (**Supplementary Table S3**). For the bta-miR-2284d in distal jejunum (down-regulated in SS, log2fold-change: −2.92), hematological system development and function (p-value < 0.05) were enriched for its negatively correlated target transcripts. Also in the duodenum, two target transcripts negatively correlated with bta-miR-2284d, MYC (MYC proto-oncogene) (*ρ* = −0.78, p-value < 0.05) and NLK (Nemo Like Kinase) (*ρ* = −0.85, p-value < 0.05), were associated with lymphoid tissue structure and development (**Supplementary Table S3**). For the bta-miR-211 (log2fold-change: 4.51) and bta-miR-29d-3p (log2fold-change: 2.23) that were up-regulated in the RAJ of SS, hematological system development and function (p-value < 0.05), immune cell trafficking (p-value < 0.05) and hematopoiesis (p-value < 0.05) were enriched for their negatively correlated target transcripts (**Table 7**). The bta-miR-18a (log2fold-change: −1.58) in the distal jejunum was negatively correlated with ITCH (Itchy E3 Ubiquitin Protein Ligase), GJA1 (Gap Junction Protein Alpha 1), IL4R (Interleukin 4 Receptor), F3 (Coagulation Factor III, Tissue Factor) and CD7 (CD7 Molecule) (*ρ*-values < −0.6, p-values < 0.05), which were associated with immune cell trafficking and humoral immune response (p-values < 0.05) (**Supplementary Table S3**). In the cecum, the negatively correlated target transcripts of bta-miR-2094 (log2fold-change: −2.72), SYK (Spleen Associated Tyrosine Kinase) and CD22 (CD22 Molecule) (*ρ*-values < −0.6, p-values < 0.05), were associated with humoral and cell-mediated immune response (p-values < 0.05) (**Supplementary Table S3**). In RAJ, the bta-miR-1271 (log2fold-change: 2.59) was negatively correlated with SCARB1 (Scavenger Receptor Class B Member 1), CNR2 (Cannabinoid Receptor 2), RALB (RAS Like Proto-Oncogene B), HBEGF (Heparin Binding EGF Like Growth Factor), DSC1 (Desmocollin 1) and SIT1 (Signaling Threshold Regulating Transmembrane Adaptor 1) (*ρ*-values < −0.6, p-values < 0.05), which were associated with humoral immune response and lymphoid tissue structure and development (p-values < 0.05) (**Supplementary Table S3**).

**Table 7:** Differentially expressed miRNAs and number of target genes negatively correlated with miRNA expression in different intestinal regions.

miRNA	# predicted targets*^a^*	# negatively correlated target genes	Enriched functions*^b^*
		Duodenum:23	hematological system development and function; hematopoiesis
		Proximal jejunum:8	cell-mediated immune response; hematological system development and function
		Distal jejunum:36	hematological system development and function; immune cell trafficking
**bta-miR-378b**	514	Cecum:2	nervous system development and function: tissue morphology
		Spiral colon:38	connective tissue development and function; organismal development
		Descending colon:3	tissue morphology; connective tissue development and function
		Rectum:5	organismal development; tissue morphology
		Duodenum: N/A**^c^**	n/a
		Distal jejunum: N/A	n/a
**bta-miR-2284j**	486	Cecum: N/A	n/a
		Spiral colon: N/A	n/a
		Rectum: N/A	n/a
		Duodenum:21	tissue morphology; cardiovascular system development and function
**bta-miR-2284d**	696	Distal jejunum:65	hematological system development and function; tissue morphology
		Spiral colon: N/A	n/a
		Rectum: N/A	n/a
**bta-miR-99a-5p**	40	Proximal jejunum:1	not enriched
		Distal jejunum:1	not enriched
**bta-miR-100**	47	Distal jejunum:1	not enriched
**bta-miR-99b**	37	Distal jejunum:1	not enriched
**bta-miR-409a**	175	Distal jejunum:5	organismal development; digestive system development and function
**bta-miR-18a**	559	Distal jejunum:16	cardiovascular system development and function; skeletal and muscular system development and function
**bta-miR-2904**	678	Cecum:7	hematopoiesis; humoral immune response
**bta-miR-451**	120	Spiral colon:3	cardiovascular system development and function; tissue development
**bta-miR-1271**	443	Rectum:8	cardiovascular system development and function; connective tissue development and function
**bta-miR-2887**	226	Rectum: N/A	n/a
**bta-miR-211**	803	Rectum:69	hematological system development and function; immune cell trafficking
**bta-miR-29d-3p**	521	Rectum:74	hematological system development and function; hematopoiesis

a Number of genes encoding miRNA target transcripts, predicted by miRanda and PITA.

b The Fisher’s exact test p-value < 0.05 calculated by Ingenuity Pathway Analysis, and the top two functions with the smallest p-values were shown. Function enrichment was not performed if the negatively correlated miRNA-mRNA pairs were less than 2.

c The correlation analysis not performed because the miRNA expressed (cpm > 1) in less than five animals.

In the distal jejunum, several negatively correlated transcripts with bta-miR-18a, bta-miR-378b, and bta-miR-2284d were also identified as DE genes between NS and SS, including the F3 (log2fold-change: 2.23) with bta-miR-18a (*ρ* = −0.67, p-value < 0.05) and with bta-miR-2284d (*ρ* = −0.85, p-value < 0.05); PTGS2 (Prostaglandin-Endoperoxide Synthase 2) (log2fold-change: 1.3) with bta-miR-2284d (*ρ* = −0.78, p-value < 0.05); THEMIS (Thymocyte Selection Associated) (log2-fold-change: 1.4), ITK (IL2 Inducible T-Cell Kinase) (log2fold-change: 1.1) and TIFA (TRAF Interacting Protein With Forkhead Associated Domain) (log2fold-change: 1.2) with bta-miR-378b (*ρ*-values were −0.73, −0.71, and −0.78 respectively, and p-values < 0.05). Also, in RAJ, the DE genes SIT1 (log2fold-change, −2.3) and RGS13 (Regulator Of G-Protein Signaling 13) (log2-fold-change, −2.2) [also reported in previous publication Wang O. et al. (2016)] were predicted as targets of bta-miR-1271 and bta-miR-29d-3p, respectively, with expression being negatively correlated with these miRNAs (ρ-values were −0.67 and −0.83 respectively, and p-values < 0.05).

## Discussion

This is the first study to investigate the potential role of miRNA in shedding of *E. coli* O157 in cattle through comparison of miRNAomes of the whole intestinal tract of SS and NS beef steers. Our findings indicated that different regions of the gut tended to share the same expressed miRNAs, and the hierarchical cluster and PCA analyses suggested similar expression patterns throughout the regions of the bovine intestinal tract. Similar results have previously been reported for miRNA expression patterns in the duodenum and jejunum of dairy cattle (Wang D. et al., 2016). The observation of high abundance of bta-miR-143 and bta-miRNA-192 was also observed in previous miRNA profiling studies of the gut tissues of calves (Liang et al., 2014; Liang et al., 2016) and dairy cattle (Wang D. et al., 2016). The bta-miR-143, which had the highest abundance in all the intestinal regions, belongs to miR-143 family, and miR-143 has been reported to be highly expressed in the gut of humans (Gaulke et al., 2014) and mice (Singh et al., 2011). This likely reflects the important function in the gut that bta-miR-143 plays in maintaining smooth muscle cells, vascular homeostasis, epithelium regeneration, and epithelial tumor repression (Slaby et al., 2007; Elia et al., 2009; Chivukula et al., 2014). Functional enrichment for the most abundant miRNAs showed associations with development of the nervous system, vascular system, and digestive system, suggesting the critical role of abundantly expressed miRNAs in the bovine gut.

Although both environmental and host related factors could influence *E. coli* O157 shedding in cattle, since both NS and SS were raised in the same environment, we speculate that the identified DE miRNAs between SS and NS are host related mechanisms which regulate gut gene expression and alter gut environment in a manner that contributes to the shedding of *E. coli* O157. The distal jejunum and RAJ were the regions where most DE miRNAs were identified, and thus it is possible that certain miRNAs expressed in these regions may be involved in *E. coli* O157 super-shedding. For example, the miRNAs, bta-miR-2284j, bta-miR-378b, and bta-miR-2284d are simultaneously down-regulated in the distal jejunum and RAJ of SS. In addition, the intestinal mucosal immune surveillance components, Peyer’s patches (PP) and isolated lymphoid follicles (ILFs) are known to be present in distal jejunum (Mutwiri et al., 1999) and RAJ (Naylor et al., 2003), respectively. Also, the attaching and effacing lesion caused by *E. coli* O157 was reported in the jejunum of challenged neonatal calves (Dean-Nystrom et al., 1997), and both innate and adaptive immune responses were detected in the RAJ of challenged cattle (Nart et al., 2008). Therefore, the miRNAs, bta-miR-2887 (down-regulated in RAJ of SS) and bta-miR-211 (up-regulated in RAJ of SS), which potentially target mRNAs involved in host immune functions, may also play a role in *E. coli* O157 shedding in SS cattle.

Down-regulation of bta-miR-378b in all the intestinal regions of SS indicates its potentially important role in *E. coli* O157 super-shedding. According to miRbase (Release 21) (Griffiths-Jones et al., 2006), bta-miR-378b is encoded by miR-378 gene family, and miRNAs of this family were suggested to regulate lipogenesis in adipose tissues (Gerin et al., 2010). Also, miR-378 was detected in monocytes and T-cells (Oertli et al., 2011) and has been reported to regulate cytotoxicity of natural killer cells (Wang et al., 2012), indicating a potential role of miR-378 in mediating innate and adaptive immune functions. Indeed, in all the intestinal regions, the immune functions were enriched for the negatively correlated target transcripts of bta-miR-378b. The PRDM1 and CYLD were two predicted target transcripts of bta-miR-378b that were also negatively correlated with bta-miR-378b in RAJ, the known primary colonization site of *E. coli* O157 in cattle (Nart et al., 2008). The PRDM1 gene encodes a Blimp1 protein, which was known to positively regulate the differentiation of T-cells into long-lived plasma cells, and it is required for the release of antibodies by these cells (Martins and Calame, 2017). Lipopolysaccharide (LPS), a byproduct of Gram-negative bacteria, was reported to cause increase in expression of PRDM1 (Savitsky and Calame, 2006). CYLD was confirmed to be a NF-*κ*B repressor to prevent inflammatory diseases and also, with the LPS being one of the factors that induces an increase in its expression (Courtois, 2008). The functions of these two transcripts in humoral and cell-mediated immune responses suggest their potential role in the host and gut microbial interactions. Down-regulation of bta-miR-378b suggested a trend of increased expression of both genes in RAJ of SS, which may be influenced by translocation of Gram-negative bacteria (as indicated above LPS may lead to altered expression of these genes), which is commonly observed when the host intestinal mucosal barrier is disrupted or immune defenses are deficient (Berg, 1999; Ryan et al., 2006). Indeed, our previous RNA-Seq based study of the rectal tissues of SS suggested possible immunodeficiency in RAJ of SS (Wang O. et al., 2016), and the capability of *E. coli* O157 to disrupt and invade cattle intestinal epithelium has also been demonstrated (Sheng et al., 2011). Future studies to measure LPS and to evaluate the abundance of Gram-negative bacteria in SS *vs* NS, as well as to examine the integrity of host intestinal mucosal barriers of SS are needed to validate the above speculation.

The bovine specific bta-miR-2284d was down-regulated in both the small and large intestine of SS, including in the RAJ. Although its expression level was low in all the tissues (average cpm = 2.2), it may still play a critical role in bovine physiology, as the number of its predicted targets is higher than that of other DE miRNAs except for bta-miR-211. Bta-miR-2284d has been reported in several bovine miRNA studies (Liu et al., 2014; Zhao et al., 2016); however, information about its function is limited. The correlation analysis suggests that this miRNA may play a regulatory function with regard to immunity in the bovine distal jejunum. Among the transcripts showing negative correlation with bta-miR-2284d in distal jejunum, the DE gene F3 (also called TF) was reported to be involved in recruiting leukocytes in the intestine of mice (Anthoni et al., 2007), and the PTGS2 gene (also called COX2) was suggested to promote humoral immune responses (Ryan et al., 2006). However, data of bacterial translocation, host mucosal integrity as well as the state of host immune defenses in the intestinal regions studied are not available, and the interaction between miR-378b/bta-miR-2284d and their target transcripts requires further validation. Nevertheless, these findings highlight the importance of further research on the role of miRNA regulation of immune functions including the intestinal mucosal barrier and immune defenses of SS cattle using histological and immunological methods, to identify miRNAs associated with host responses against *E. coli* O157. Also, to better understand the role of bta-miR-378b in super-shedding phenomena, it is worth further investigating whether it directly targets the genes associated with the immune system, or regulates immune functions through regulating lipid metabolism, as lipids are important components for cell signaling and cell proliferation involved in immune responses (Bensinger et al., 2008). In addition, to validate the interaction between bta-miR-2284d and potential targets, especially the targets involved in immune functions, will help understand the functions of this bovine specific miRNA, and how its regulation may influence *E. coli* O157 shedding in cattle.

A recent report suggested that fecal miRNAs were capable of maintaining normal gut microbiota (Liu et al., 2016) by entering bacteria cells including *Fusobacterium nucleatum* (*F. nucleatum*) and *E. coli*, to influence bacterial gene expression possibly through interaction with bacterial DNA/RNA (Liu et al., 2016). Although it is unclear whether the DE miRNAs reported in this study serve functions in the intestinal luminal spaces and their capability of entering bacterial cells including *E. coli* O157 are unknown; these miRNAs are worth further study as it is necessary to show the direct interaction with gut microbes and influence of *E. coli* O157 shedding in cattle.

One of the limitations of the current study is that the animals were not traced for shedding levels to determine whether they are transient shedders or persistent shedders. However, the miRNAs that may play a role in regulating the genes which influence the *E. coli* O157 shedding in cattle may serve as the candidates for future validation in larger population and for different ages of the animals.

In conclusion, this study investigated the miRNA expression in the whole intestine of *E. coli* O157 SS and NS beef steers. The miRNA profiling results indicated that the majority of expressed miRNAs were common within the regions of the intestinal tract that were investigated. Comparative expression analysis of miRNAs revealed that the distal jejunum, and RAJ may play an important role in host responses against *E. coli* O157, as most DE miRNAs between SS and NS were identified in these regions. Down-regulation of bta-miR-378b and bta-miR-2284d in multiple intestinal regions of SS suggested that these two miRNAs may differentially alter lipid metabolism and immune functions in the intestinal tract of in the SS vs NS, influencing host and *E. coli* O157 interactions and leading to its super-shedding in cattle. The identified miRNAs and their functions provide better understanding of the molecular mechanisms that regulating colonization of this foodborne pathogen *in vivo*, which is vital for development of better strategies to reduce cattle super shedding of *E. coli* O157.

## Data Availability Statement

The miRNA-Seq data are available at NCBI Gene Expression Omnibus (GEO) database under accession number GSE96973.

## Ethics Statement

The steers used in this experiment followed the Canadian Council of Animal Care Guidelines, and the protocol was reviewed and approved by the Animal Care Committee of Lethbridge Research Centre, Agriculture Agri-food Canada (Animal Care Committee protocol number: 1120; approved June 2011).

## Author Contributions

OW and LG conceived and designed the experiments. OW, MZ, YC, TM, and BS performed the experiments. OW, MZ, and LG analyzed the data. LG contributed reagents/materials/analysis tools. OW, MZ, TM, GP, KS, BS, and LG wrote and edited the paper. All authors contributed to the article and approved the submitted version.

## Funding

This research was funded by Alberta Innovates Bio Solutions (Project Number: FSC-12-017) and Beef Cattle Research Council (BCRC, FOS.07.17).

## Conflict of Interest

The authors declare that the research was conducted in the absence of any commercial or financial relationships that could be construed as a potential conflict of interest.

## Supplementary Material

Supplementary Figure 1 Results of Euclidean distance based hierarchical clustering and PCA analysis based on miRNA expression profiles of each intestinal regions.
