# Supplementary figures and images for: MicroRNAomes of Cattle Intestinal Tissues Revealed Possible miRNA Regulated Mechanisms Involved in Escherichia coli O157 Fecal Shedding
Source: Front Cell Infect Microbiol. 2021 Feb 24;11:634505. doi: 10.3389/fcimb.2021.634505 (PMC7959717; doi:10.3389/fcimb.2021.634505)

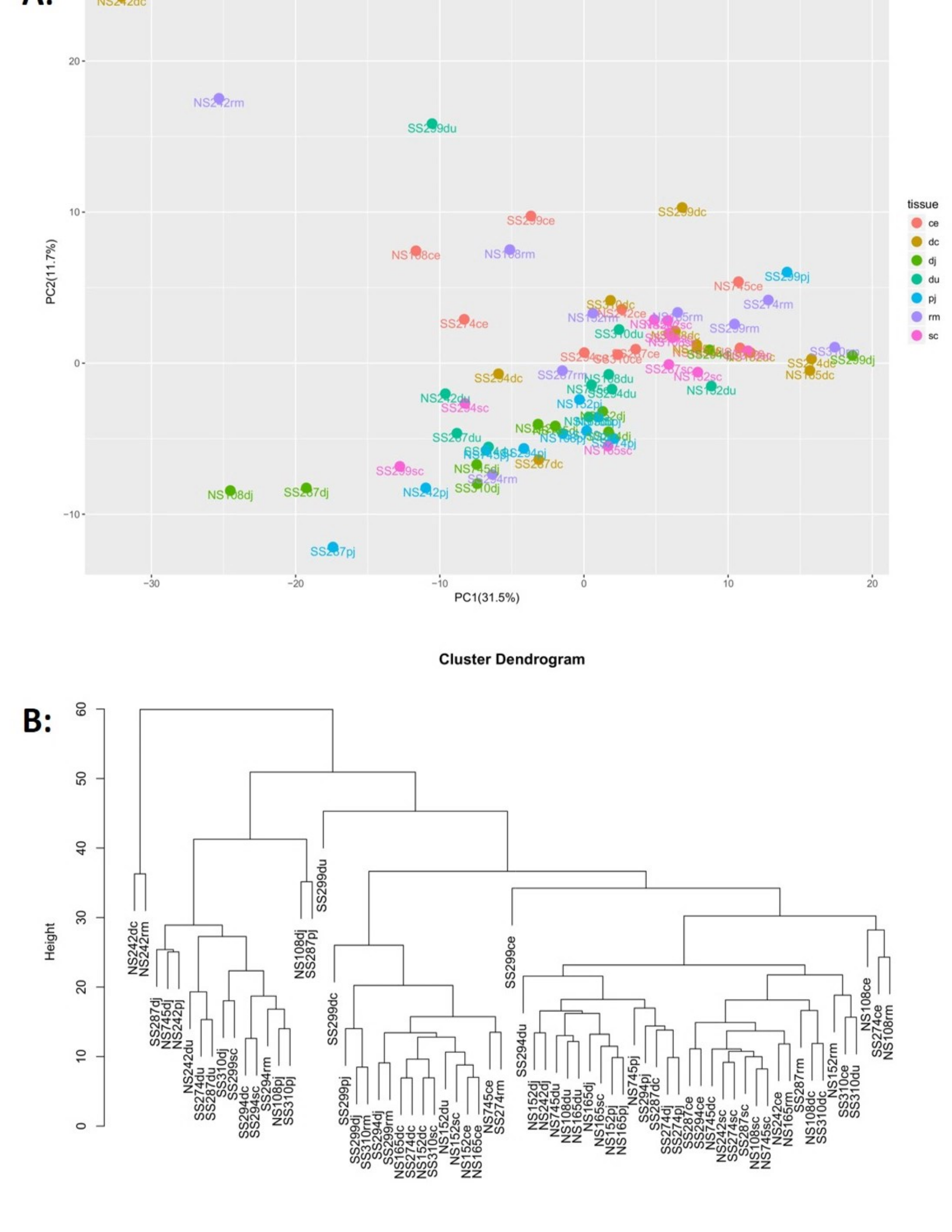

Supplement: Supplementary Figure 1 — Results of Euclidean distance based hierarchical clustering and PCA analysis based on miRNA expression profiles of each intestinal regions. [file Image_1.tiff]
