# Supplementary material for: MicroRNAomes of Cattle Intestinal Tissues Revealed Possible miRNA Regulated Mechanisms Involved in Escherichia coli O157 Fecal Shedding
Source: Front Cell Infect Microbiol. 2021 Feb 24;11:634505. doi: 10.3389/fcimb.2021.634505 (PMC7959717; doi:10.3389/fcimb.2021.634505)
Supplement: Supplementary file 2 [file Table_1.docx]

**Supplementary Table S1**^#^**.** *Escherichia coli* O157:H7 numbers (log_10_ CFU/g feces)* of the tested samples.

| **Steer ID** | ***E. coli* O157:H7 numbers at the day of sampling,**  **log CFU/g feces** | **Length of time between the first sampling and the day of slaughter, days** | ***E. coli* O157:H7 numbers day of/prior to slaughter,**  **log CFU/g feces** |
| --- | --- | --- | --- |
| **274_SS** | 6.7 | 8 | +** (a day prior to slaughter) |
| **287_SS** | 5.4 | 8 | + (a day prior to slaughter) |
| **294_SS** | 5.8 | 10 | + (a day prior to slaughter) |
| **299_SS** | 7.8 | 10 | + (a day prior to slaughter) |
| **310_SS** | 7.5 | 4 | 5.8 (day of slaughter) |

^#^Adapted and revised from Wang et al. (2016).

**E. coli* O157:H7 number in fecal samples of steers identified as super-shedders at the day of sampling and day of/prior to slaughter.

**+, positive for *E. coli* O157:H7 via immunomagnetic separation assay.
